# Supplementary material for: FIORA: Local neighborhood-based prediction of compound mass spectra from single fragmentation events
Source: Nat Commun. 2025 Mar 7;16:2298. doi: 10.1038/s41467-025-57422-4 (PMC11889238; doi:10.1038/s41467-025-57422-4)
Supplement: Supplementary file 1 — Supplementary Information [file 41467_2025_57422_MOESM1_ESM.pdf]

Supplementary Information

FIORA: Local neighborhood-based prediction of compound mass spectra from single fragmentation events

Yannek Nowatzky [1,2] Francesco Friedrich Russo,[3,4] Jan Lisec [3] Alexander Kister,[1] Knut Reinert [2,5] Thilo Muth [2,6] and Philipp Benner [1,*]

[1] Section VP.1 eScience, Federal Institute for Materials Research and Testing (BAM), Berlin, Germany
[2] Department of Mathematics and Computer Science, Freie Universität Berlin, Berlin, Germany
[3] Department of Analytical Chemistry and Reference Materials, Organic Trace Analysis and Food Analysis, Federal Institute for Materials Research and Testing (BAM), Berlin, Germany
[4] Institute of Pharmacy, Freie Universität Berlin, Berlin, Germany
[5] Department of Computational Molecular Biology, Max Planck Institute for Molecular Genetics, Berlin, Germany
[6] Data Competence Center MF 2, Robert Koch Institute, Berlin, Germany
[*] Lead Contact and correspondence: philipp.benner@bam.de

CONTENTS

Supplementary Figures 3

Supplementary Tables 20

Supplementary Results 21

Summary . . . 21
Cosine similarity: Distributions and different flavors . . . 21
Adducts . . . 22
The impact of collision energy . . . 22
Data analysis for CASMI 22 . . . 23
Mathematical aspects . . . 24

LIST OF FIGURES

1 Illustration of a graph network over multiple graph convolutions. . . . 3
2 Spectral mirror plot for *Indole-3-acetyl-L-alanine* in **positive $[M+H]^+$** ionization mode. . . . 4
3 Spectral mirror plot for *Indole-3-acetyl-L-alanine* in **negative $[M-H]^-$** ionization mode. . . . 4
4 Cosine similarity distribution of test predictions with and without precursor. . . . 5
5 Histogram of cosine similarity split according to test set. . . . 6
6 MS/MS spectra of SNS-032 acquired using a low to moderate collision energy of 20 eV . . . 6
7 MS/MS spectra of SNS-032 acquired using a moderate collision energy of 30 eV . . . 7
8 MS/MS spectra of SNS-032 acquired using a high collision energy of 60 eV . . . 8
9 Cosine similarity distribution at collision energy intervals. . . . 9
10 Peak intensity coverage at collision energy intervals. . . . 9
11 Cosine similarity distribution without the precursor peak at collision energy intervals. . . . 10
12 Cosine similarity between the CASMI 16 challenge spectra and FIORA's predictions at collision energy levels 20, 35, 50 (NCE), and the merged prediction. . . . 11
13 A comparison of cosine similarity of CASMI 22 at collision energy levels 35, 45, 60 (NCE). . . . 11

14 A comparison of cosine similarity without precursor of CASMI 22 at collision energy levels 35, 45, 60 (NCE). 12
15 Distribution of MS/MS characteristics in the test datasets. . . . 13
16 Histogram showing structural similarity of test compounds to the training compounds. . . . 14
17 Cosine similarity without the precursor peak at intervals of structural similarity of test compounds to training compounds. . . . 14
18 Global UMAP of graph embeddings depicting *lipids and lipid-like molecules* annotated at the compound class level (global arrangement) . . . 15
19 Local UMAP of graph embeddings depicting *lipids and lipid-like molecules* annotated at the compound class level (local arrangement) . . . 16
20 Distributions of different similarity scores and their biases evaluated on the test split. . . . 17
21 Recall at k . . . 18
22 Recall at k without the precursor peak . . . 18
23 Recall at k for different cosine scores . . . 19
24 Grid search results showing interquartile ranges . . . 19

LIST OF TABLES

1 Median cosine similarity without precursor peak. . . . 20
2 Median cosine similarity without any transformation. . . . 20
3 Median cosine similarity of [M]+ and [M]- predictions to ground truth spectra from the MSnLib test split. . . . 20
4 Summary of molecular features, model specifications and training parameters. . . . 20
5 Overview of the spectral libraries used for training. Sources list only the biggest contributions. Additional information is found on the provider websites `https://www.sisweb.com/software/ms/NIST’17.pdf` `https://systemsomicslab.github.io/compms/msdial/main.html`, and `https://doi.org/10.5281/zenodo.11163381` [5–7]. 21

SUPPLEMENTARY FIGURES

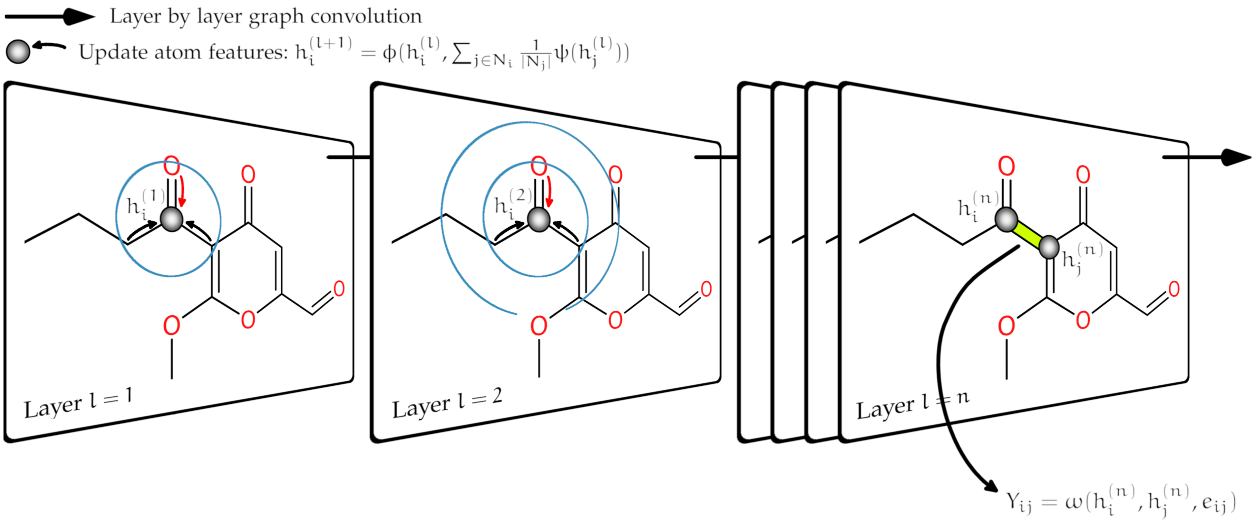

Supplementary Figure 1: Illustration of a graph network over multiple graph convolutions.
Illustration of how a graph network translates local structure information into molecular property prediction. The network performs multiple graph convolutions on the molecular structure graph, thereby aggregating the local neighborhood, i.e., the surrounding substructure, into hidden representations of the atoms. Subsequently, edge prediction is used to estimate bond properties, such as their stability.

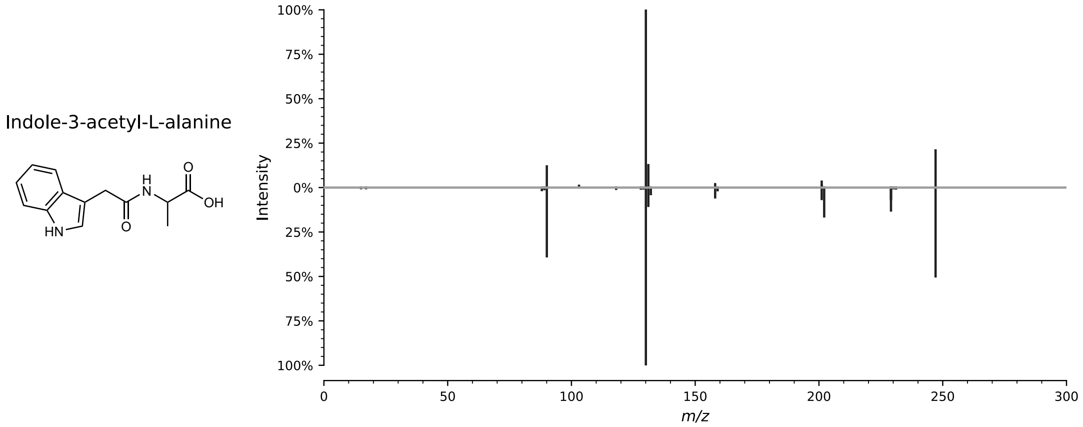

Supplementary Figure 2: Spectral mirror plot for *Indole-3-acetyl-L-alanine* in positive [M+H]$^+$ ionization mode.

Indole-3-acetyl-L-alanine is used to illustrate Fiora's fragmentation algorithm in the main manuscript. The upper panel displays the experimental spectrum from the MS-Dial library, while the lower panel shows Fiora's prediction. The cosine similarity between the two spectra is 0.92, and the maximum Tanimoto similarity to the training compounds is 0.73 (using a 2048-bit Morgan fingerprint with a radius of 3).

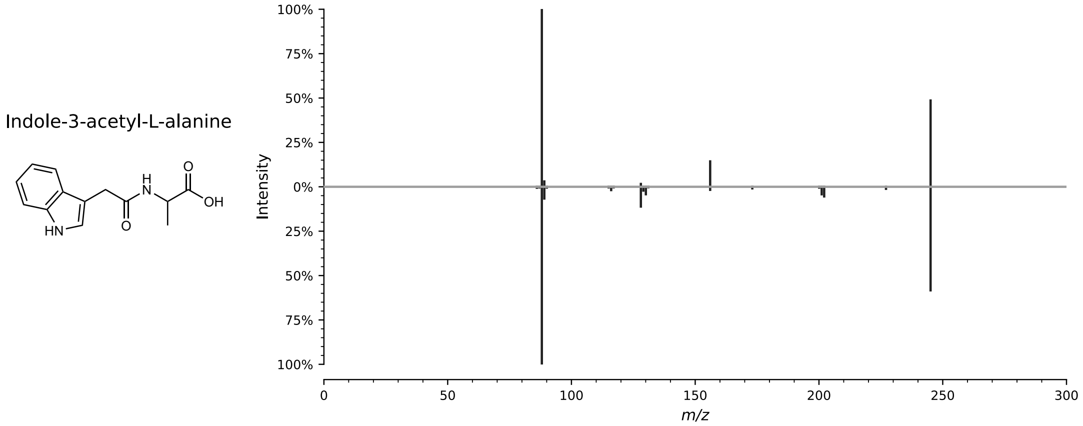

Supplementary Figure 3: Spectral mirror plot for *Indole-3-acetyl-L-alanine* in negative [M-H]$^-$ ionization mode.

Indole-3-acetyl-L-alanine is used to illustrate Fiora's fragmentation algorithm in the main manuscript. The upper panel displays the experimental spectrum from the MS-Dial library, while the lower panel shows Fiora's prediction. The cosine similarity between the two spectra is 0.98, and the maximum Tanimoto similarity to the training compounds is 0.73 (using a 2048-bit Morgan fingerprint with a radius of 3).

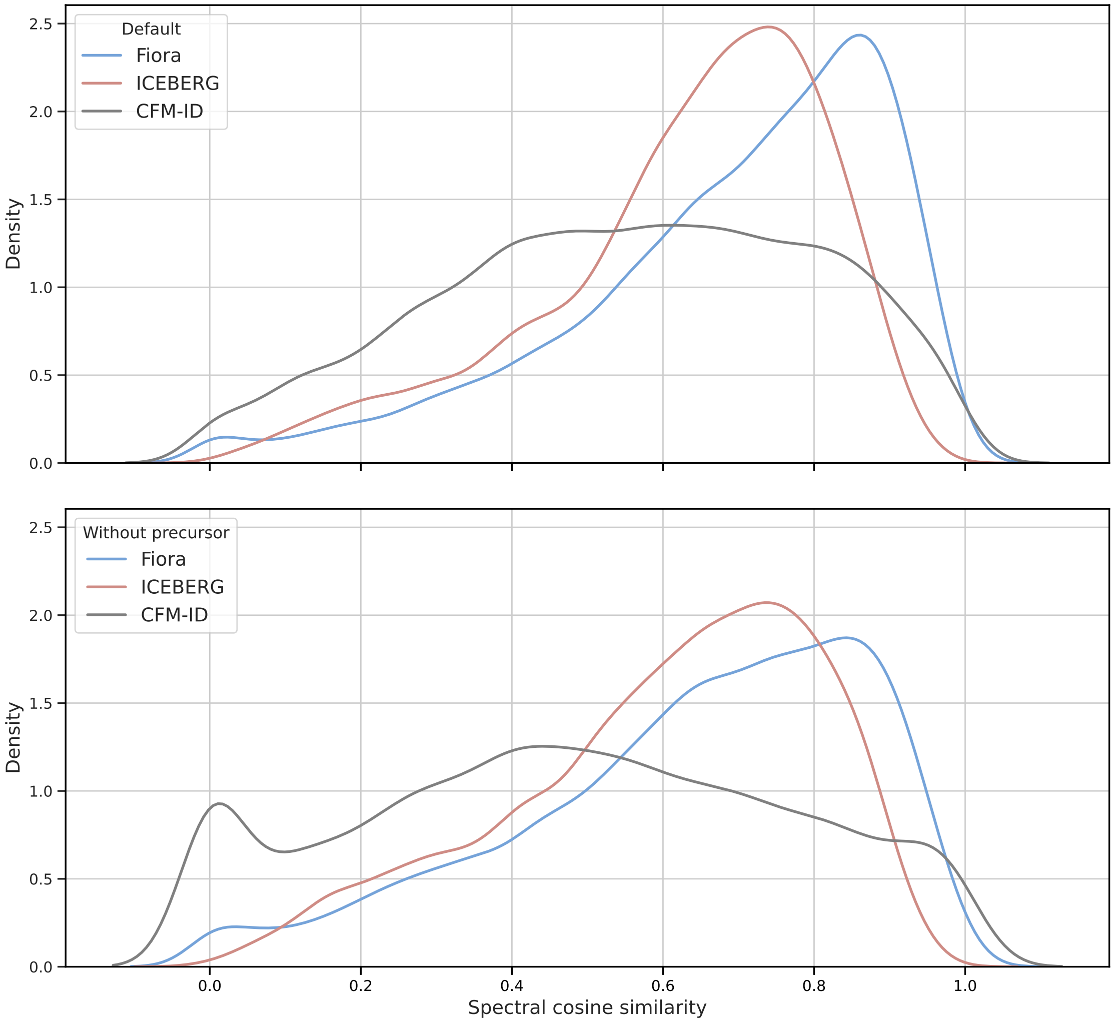

Supplementary Figure 4: Cosine similarity distribution of test predictions with and without precursor. The notable shift to the right of FIORA's curve, compared to that of ICEBERG, indicates consistently higher scores by FIORA. Both tools show a culmination of high scoring spectra (with cosine similarity of >0.6). In contrast, CFM-ID exhibits a more even spread of cosine scores. Without the precursor peaks, all tools attain lower cosine scores, observed by the curve shift to the left, but the advantage of FIORA remains.

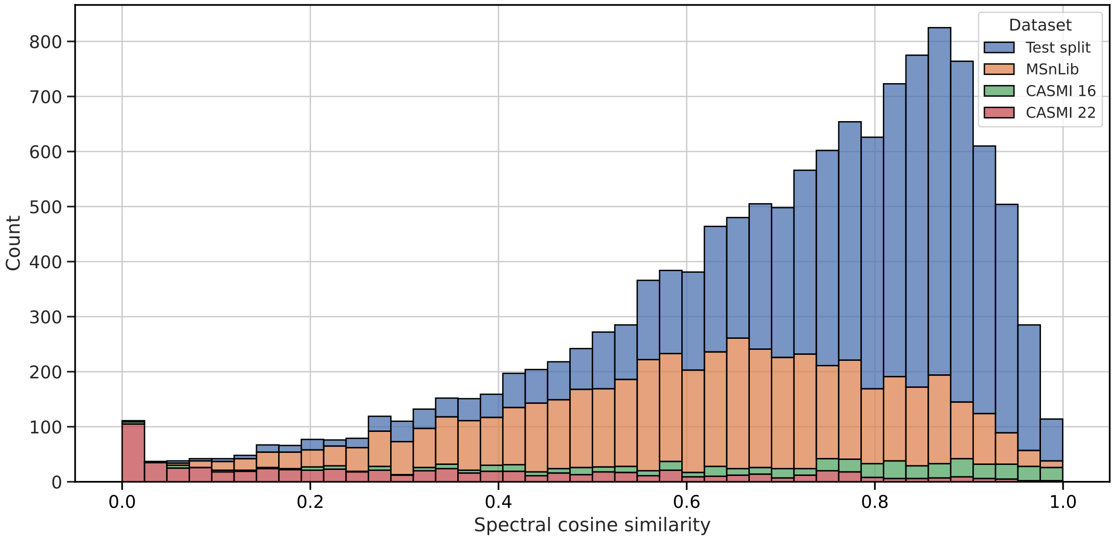

Supplementary Figure 5: Histogram of cosine similarity split according to test set.
Test split and CASMI 16 spectra have predominantly high scores of 0.8 and above. For MSnLib spectra, cosine scores are more evenly distribution with a peak at 0.6 to 0.8, while CASMI 22 scores are overwhelming low.

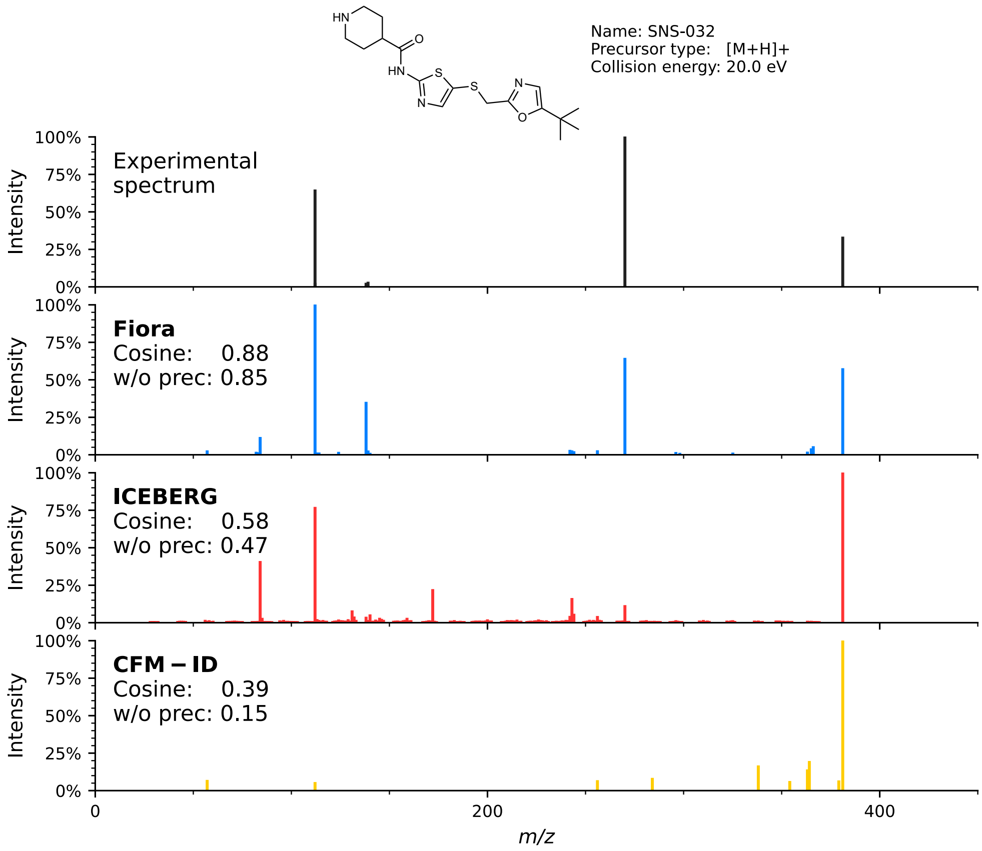

Supplementary Figure 6: MS/MS spectra of SNS-032 acquired using a low to moderate collision energy of 20 eV.
On top, the experimental ground-truth spectra is displayed; below predicted spectra of Fiora (blue), ICEBERG (red) and CFM-ID (yellow) are presented.

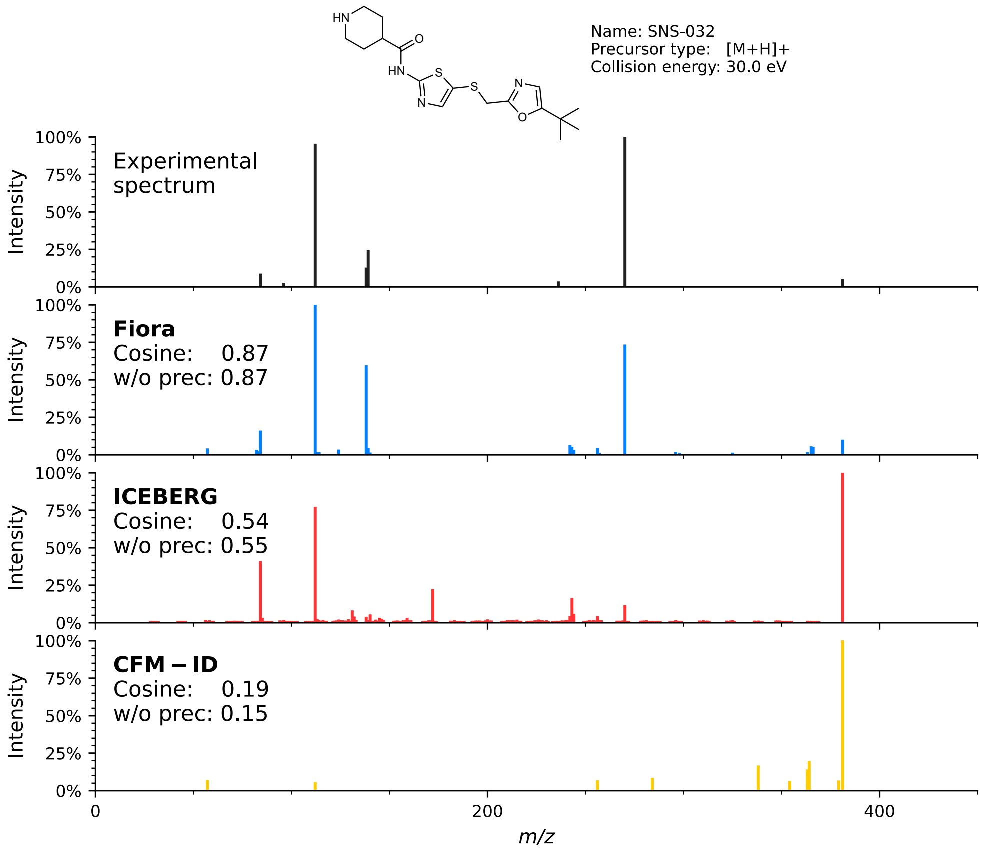

Supplementary Figure 7: MS/MS spectra of SNS-032 acquired using a moderate collision energy of 30 eV. On top, the experimental ground-truth spectrum is displayed; below predicted spectra of Fiora (blue), ICEBERG (red) and CFM-ID (yellow) are presented.

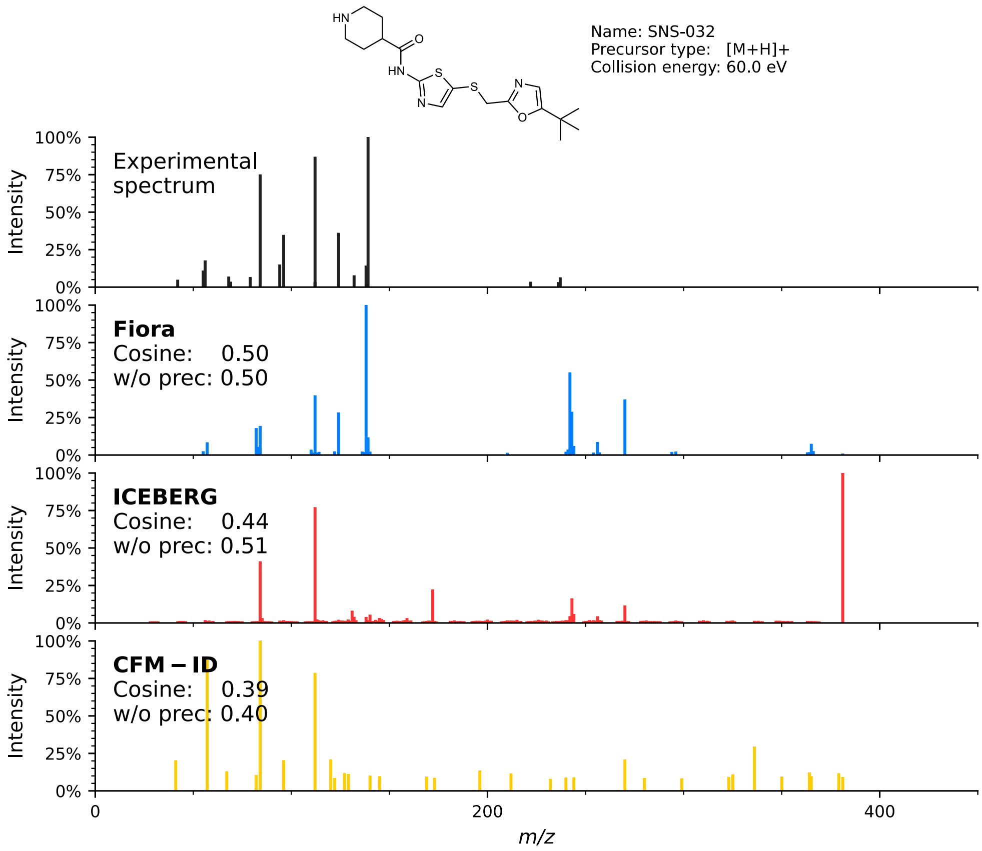

Supplementary Figure 8: MS/MS spectra of SNS-032 acquired using a high collision energy of 60 eV. On top, the experimental ground-truth spectrum is displayed; below predicted spectra of FIORA (blue), ICEBERG (red) and CFM-ID (yellow) are presented.

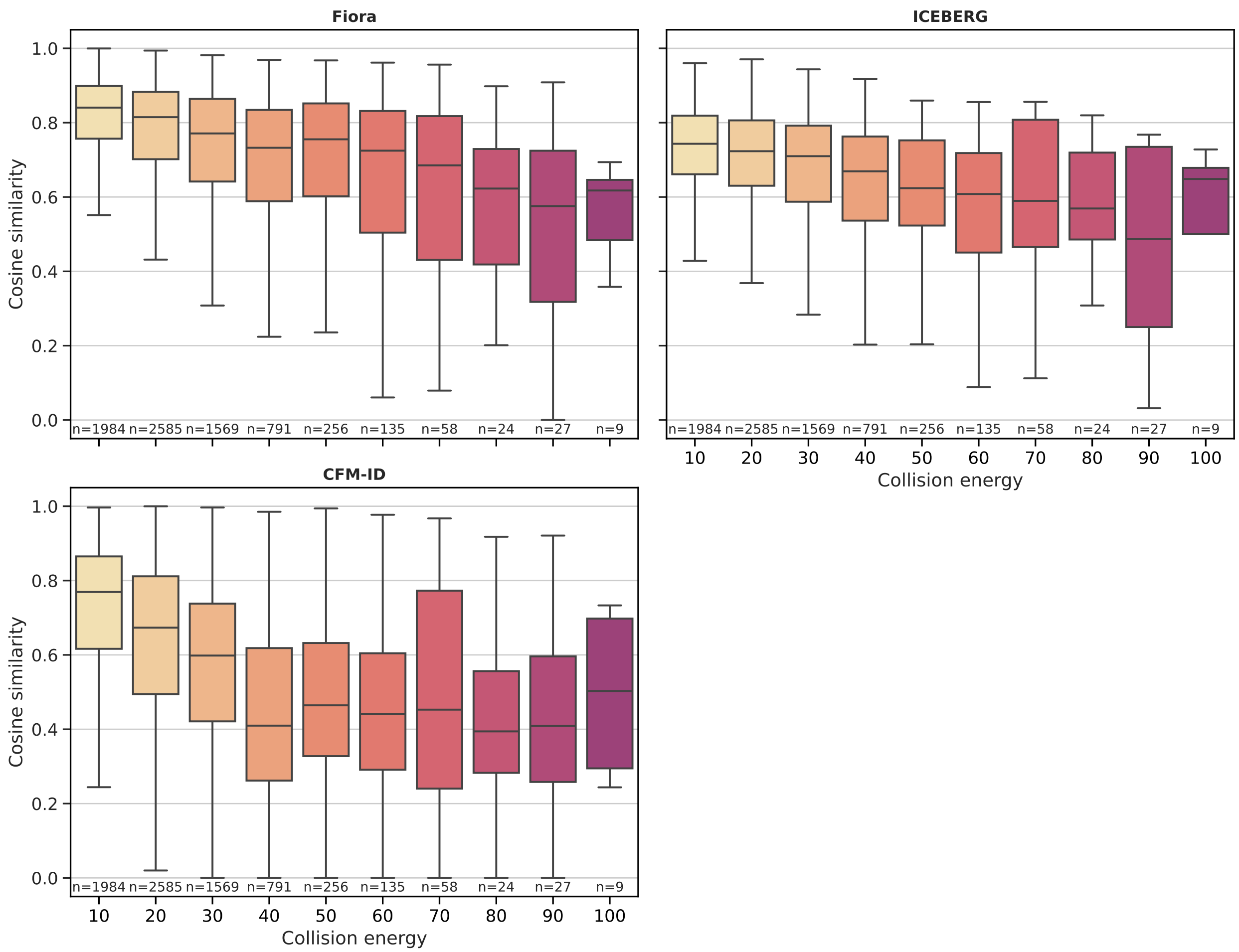

Supplementary Figure 9: Cosine similarity distribution at collision energy intervals.
The boxes represent the interquartile ranges, with the median at the centre, and the whiskers extend to the limits of the distribution (excluding outliers). For all algorithms, a decline in prediction performance with increasing collision energy can be observed.

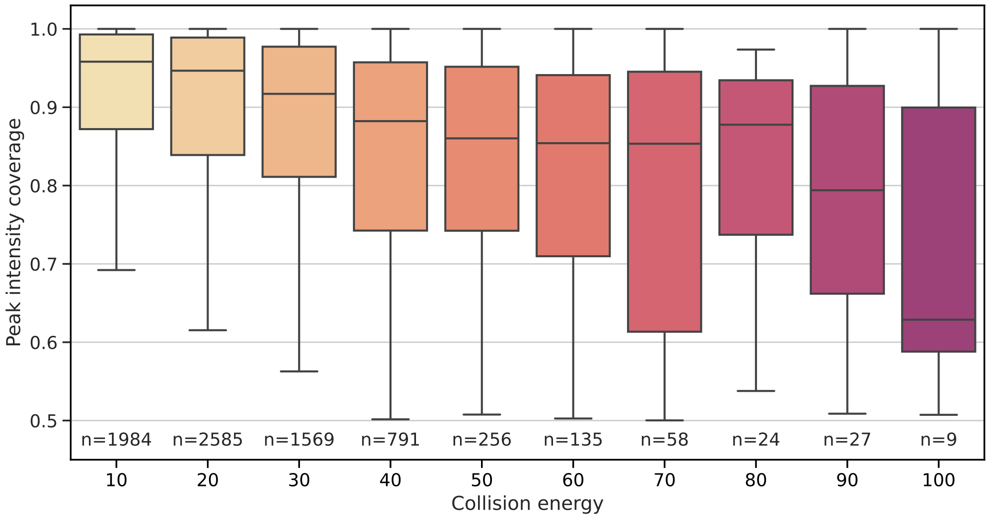

Supplementary Figure 10: Peak intensity coverage at collision energy intervals.
The boxes represent the interquartile ranges, with the median at the centre, and the whiskers extend to the limits of the distribution (excluding outliers). Coverage declines with increasing collision energy.

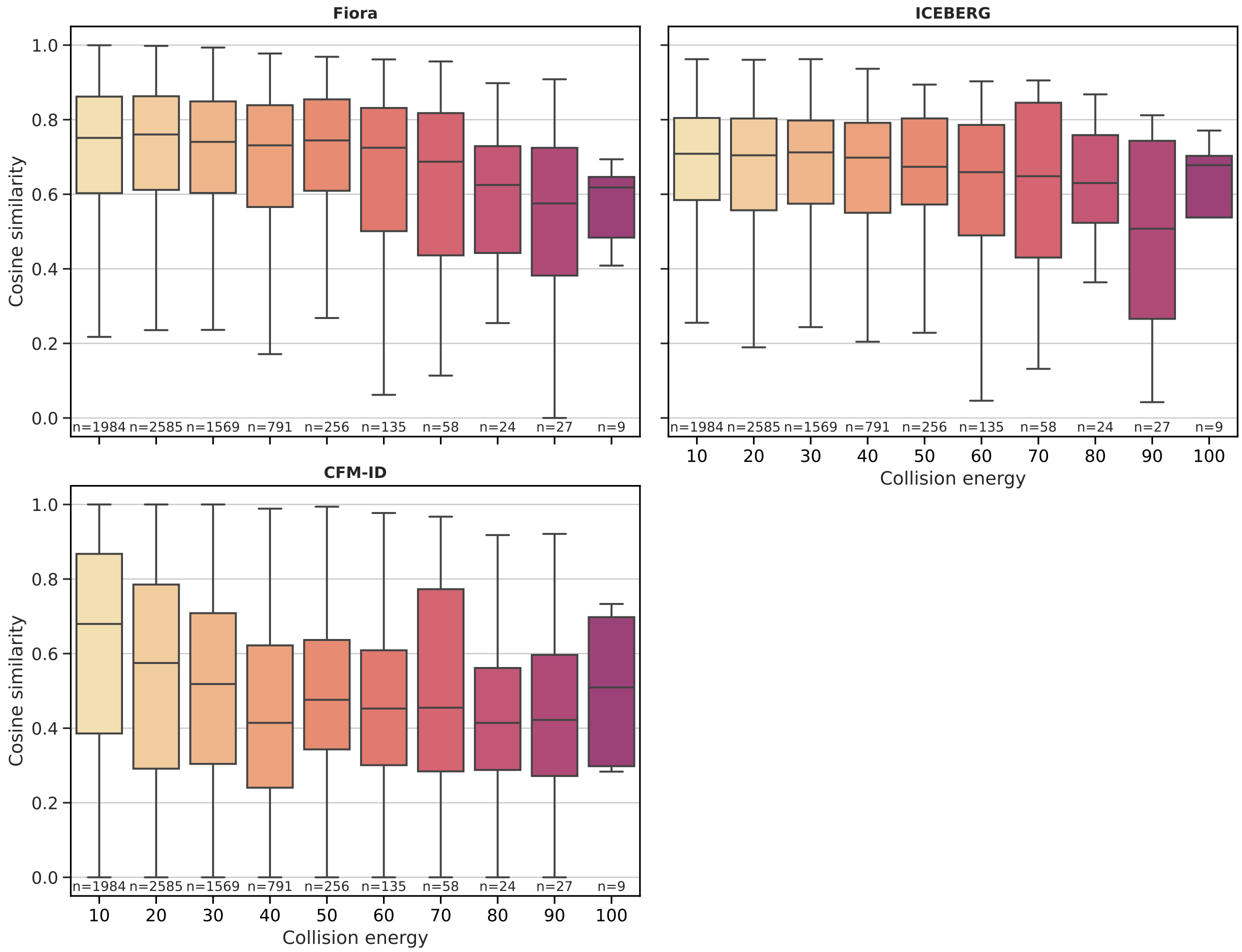

Supplementary Figure 11: Cosine similarity distribution without the precursor peak at collision energy intervals.

The boxes represent the interquartile ranges, with the median at the centre, and the whiskers extend to the limits of the distribution (excluding outliers). For all algorithms, a decline in prediction performance with increasing collision energy can be observed.

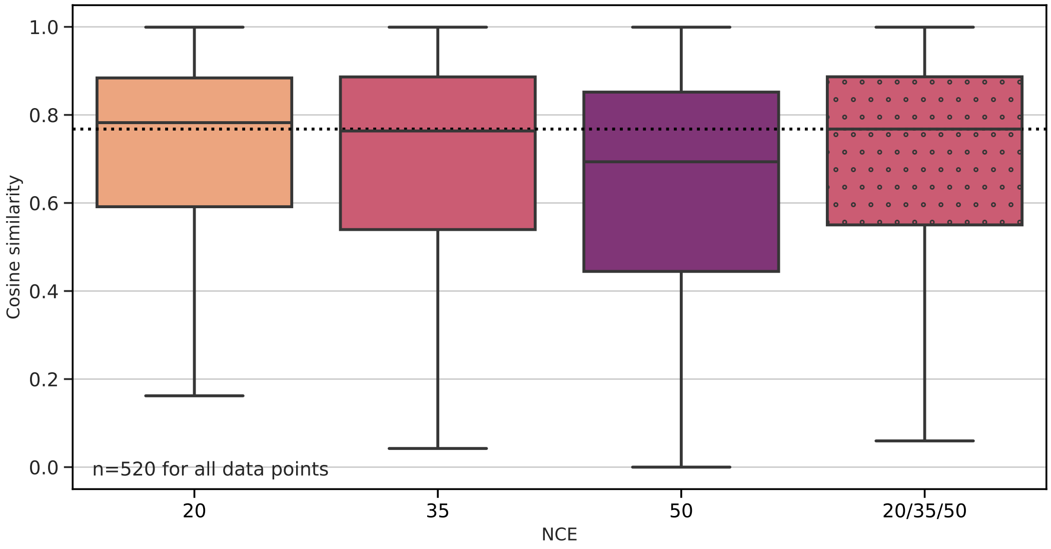

Supplementary Figure 12: Cosine similarity between the CASMI 16 challenge spectra and the predictions at collision energy levels 20, 35, 50 (NCE), and the merged prediction (highlighted).
The boxes represent the interquartile ranges, with the median at the centre, and the whiskers extend to the limits of the distribution (excluding outliers). Merging the predicted spectra of the individual energy steps, which models the experimental setup more closely, yields a slightly higher median cosine similarity than predictions at the average energy level (compare medians with the dotted line).

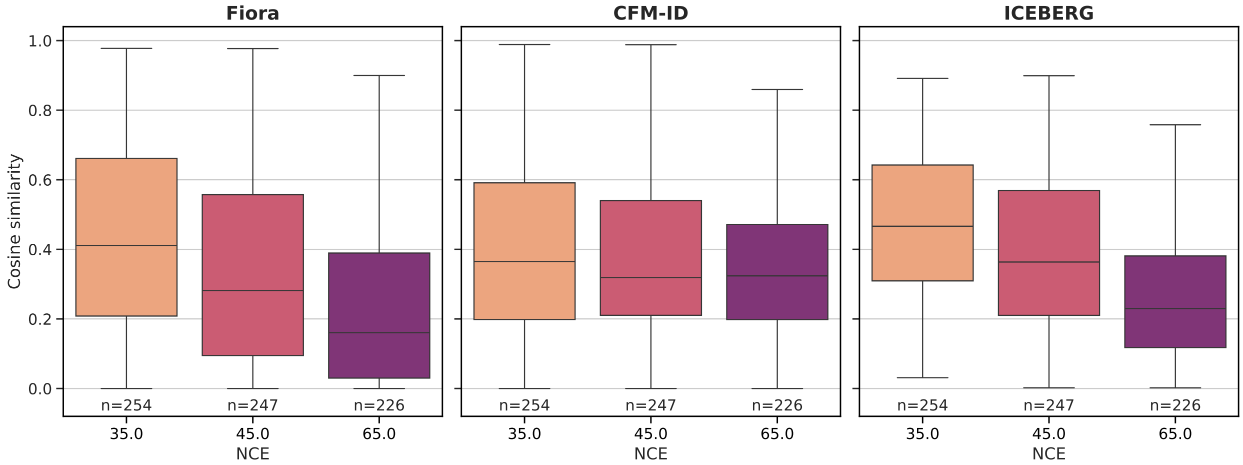

Supplementary Figure 13: A comparison of cosine similarity of CASMI 22 at collision energy levels 35, 45, and 60 (NCE).
The boxes represent the interquartile ranges, with the median at the centre, and the whiskers extend to the limits of the distribution (excluding outliers). All algorithms exhibit higher prediction quality at the lower collision energies. This trend is more pronounced for FIORA and barely visible for CFM-ID. At a normalized collision energy of 35, the performance of FIORA is comparable to that of the other algorithms and declines only at the higher energy levels.

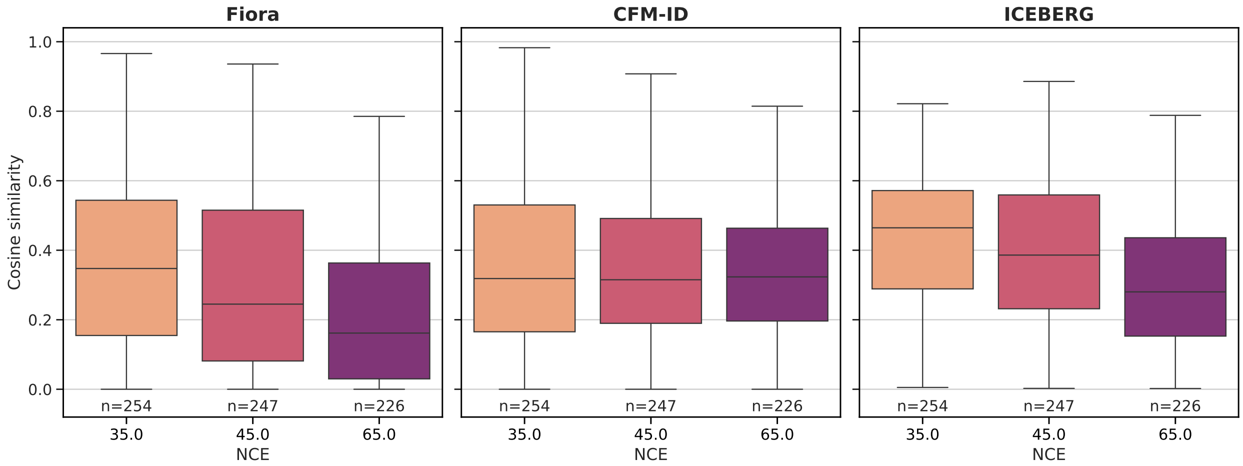

Supplementary Figure 14: A comparison of cosine similarity without precursor of CASMI 22 at collision energy levels 35, 45, and 60 (NCE).

The boxes represent the interquartile ranges, with the median at the centre, and the whiskers extend to the limits of the distribution (excluding outliers). All algorithms exhibit higher prediction quality at the lower collision energies. This trend is more pronounced for FIORA and barely visible for CFM-ID. At a normalized collision energy of 35, the performance of FIORA is comparable to that of the other algorithms and declines only at the higher energy levels.

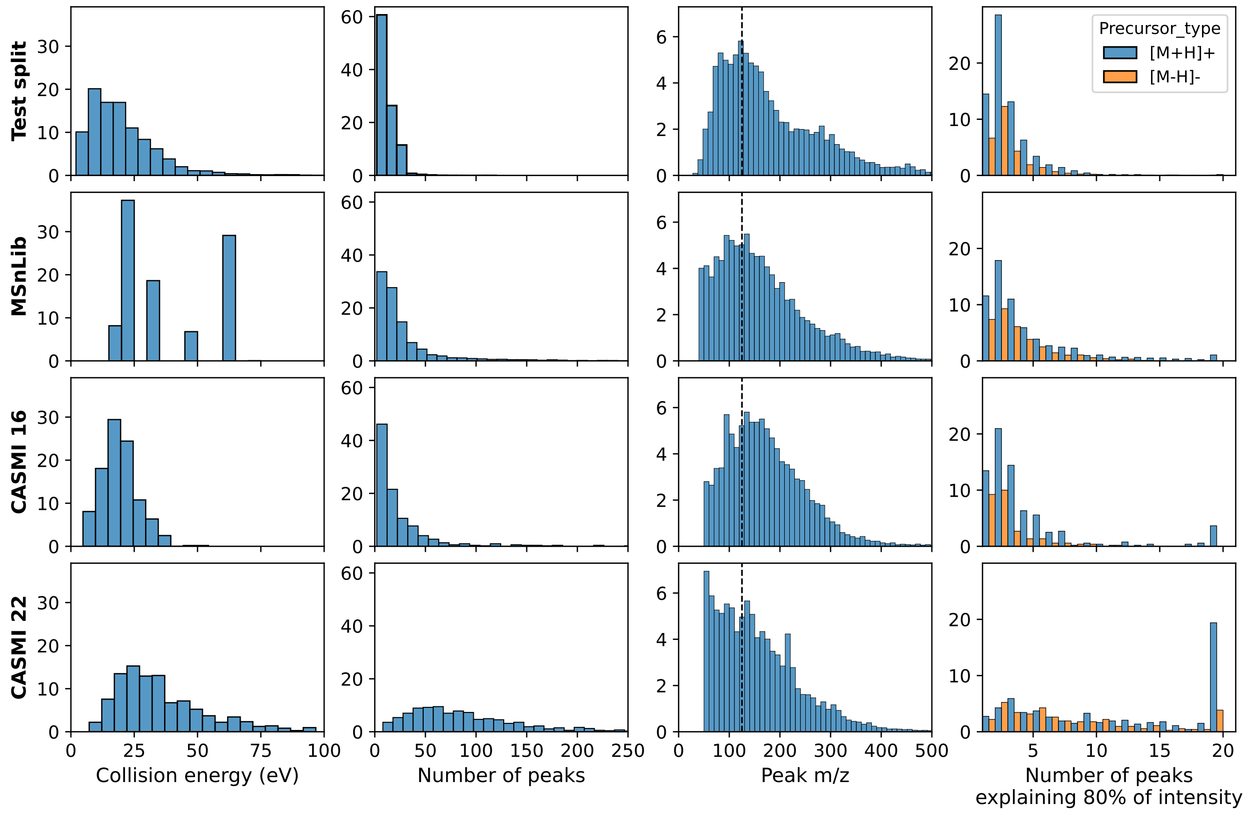

Supplementary Figure 15: Distribution of MS/MS characteristics in the test datasets.
The CASMI 22 dataset exhibits distinctively different distributions compared to other test sets. Collision energies are higher with a larger spread, the spectra contain significantly more peaks with an abundance of low m/z peaks (under 120 m/z). Lastly, peak intensities in CASMI 22 are distributed across a large number peaks, with 80% of peak intensity being explained by 20 or more peaks in many cases.

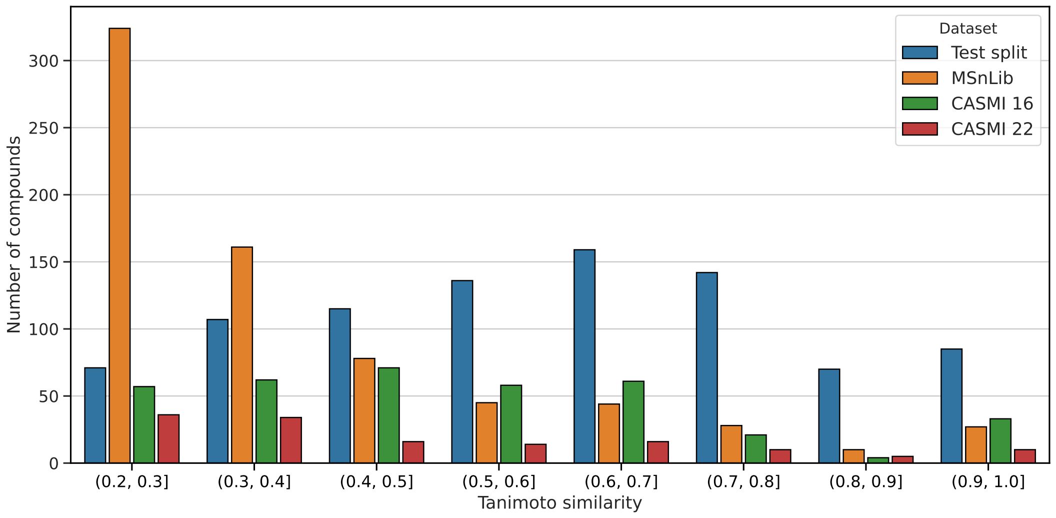

Supplementary Figure 16: Histogram showing structural similarity of test compounds to the training compounds.

Structural similarity was measured by the maximum Tanimoto similarity (Jaccard index) using Morgan fingerprints with 2048 bits and a radius of 3. Number of compounds are shown for each interval split by test sets. Overtly, MSnLib and CASMI 22 have a higher number of structurally distinct compounds (low Tanimoto similarity) than structurally similar compounds (high Tanimoto similarity). The test split and CASMI 16 exhibit a more uniform distribution, with a slight increase in compound numbers with Tanimoto similarity of and around 0.5.

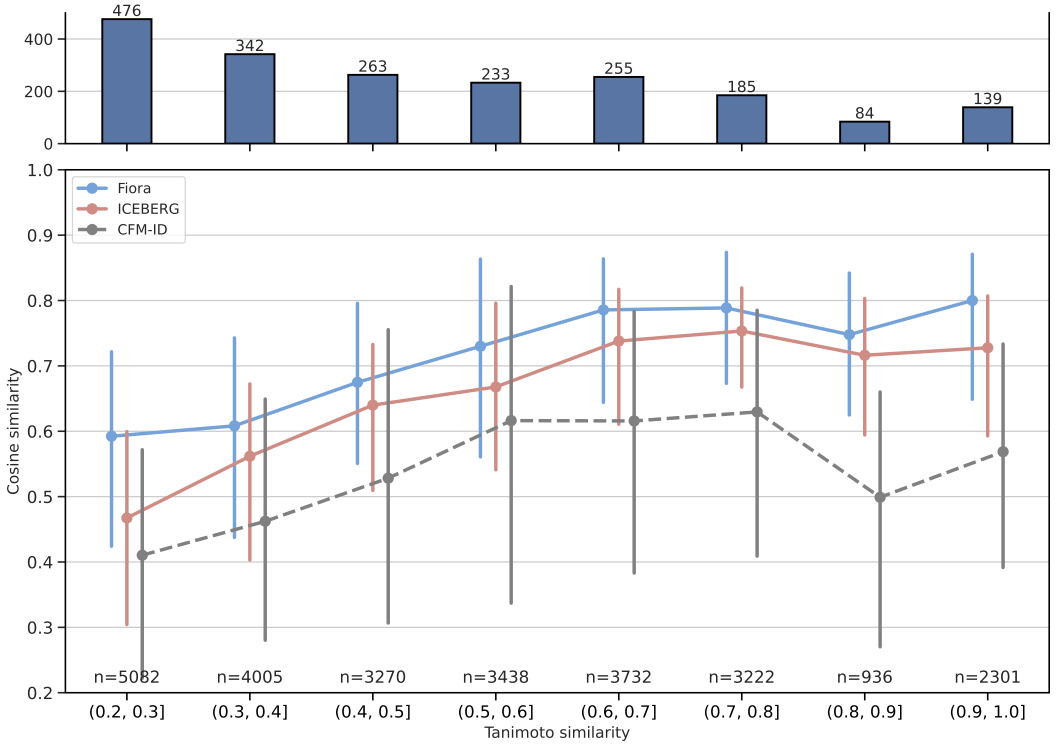

Supplementary Figure 17: Cosine similarity without the precursor peak at intervals of structural similarity of test compounds to training compounds.

Structural similarity was measured by the maximum Tanimoto similarity (Jaccard index) using Morgan fingerprints with 2048 bits and a radius of 3. The data points present the median cosine similarity and error bars denote the interquartile ranges. On the top panel, the number of unique compounds for each group is shown.

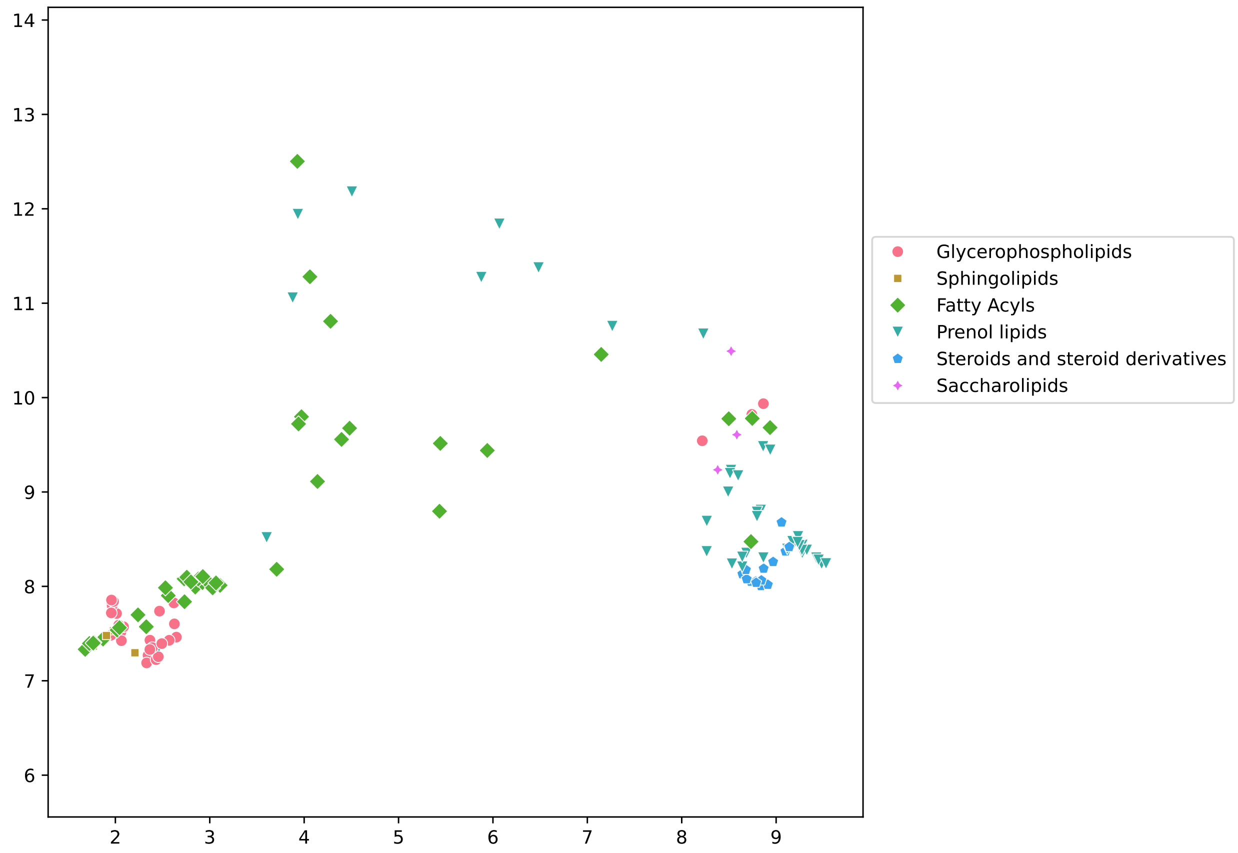

Supplementary Figure 18: Global UMAP of graph embeddings depicting *lipids and lipid-like molecules* annotated at the compound class level.

Each point corresponds to a unique compound and is colored according to compound class, which were annotated by ClassyFire [1]. Dimensionality reduction was performed with respect to all compounds, resulting in a global arrangement of compound embeddings (compared to Supplementary Figure 19, which depicts a more local representation). Note that the UMAP is not identical to the one presented in the main manuscript, in spite of using the same seed for dimensionality reduction. Compounds from other superclasses were excluded to highlight specifically the arrangement within the *lipids and lipid-like molecules*.

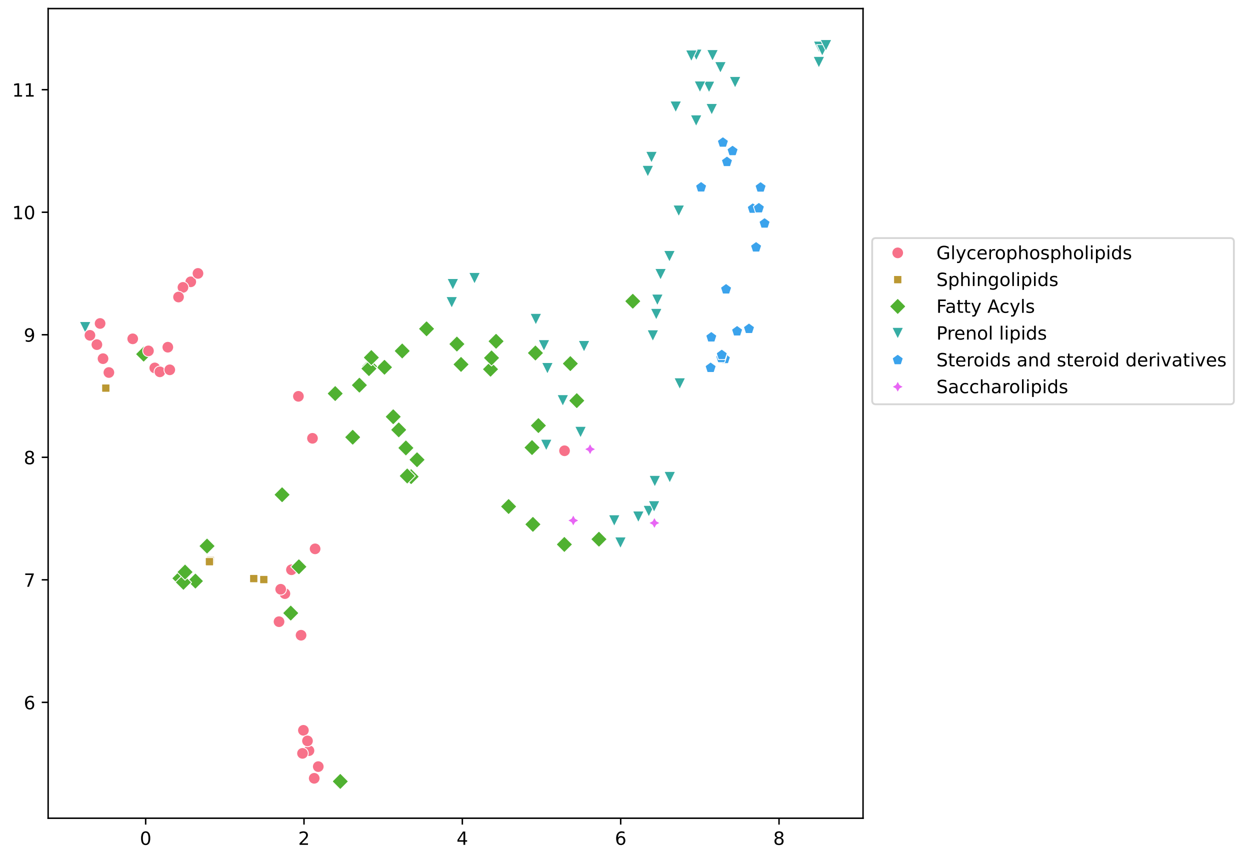

Supplementary Figure 19: Local UMAP of graph embeddings depicting *lipids and lipid-like molecules* annotated at the compound class level.

Each point corresponds to a unique compound and is colored according to compound class, which were annotated by ClassyFire [1]. Dimensionality reduction was performed considering only compound from this superclass. This illustrates a more local arrangement of compound embeddings with respect to other *lipids and lipid-like molecules* (compared to Supplementary Figure 18, which depicts a more global representation).

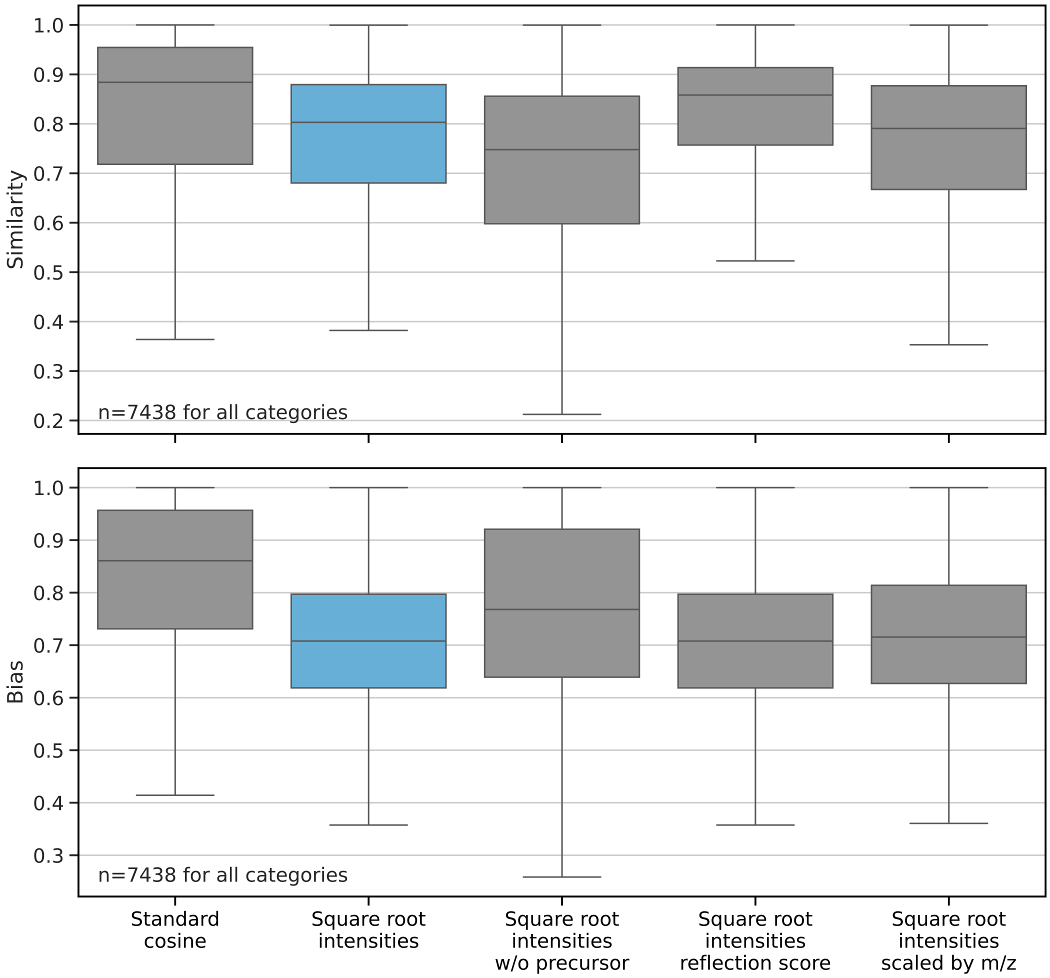

Supplementary Figure 20: Distributions of different similarity scores and their biases evaluated on the test split.

The boxes represent the interquartile ranges, with the median at the centre, and the whiskers extend to the limits of the distribution (excluding outliers). Cosine similarity using square root intensities is highlighted because it is the widely preferred spectral similarity score. It also exhibits lower bias values than the standard cosine similarity. As such, it is presumably better at capturing low-intensities peak patterns. Variants of the cosine score include the removal of the precursor peak (w/o precursor), the removal of unmatched (noise) peaks from the query (reflection score), and intensity values scaled by their m/z values. Note that some of the variants exhibit higher cosine biases (e.g., w/o precursor) or have a narrower range of similarity values (e.g., reflection score). Refer to references [2–4] for further discussions on spectral similarity scores and biases.

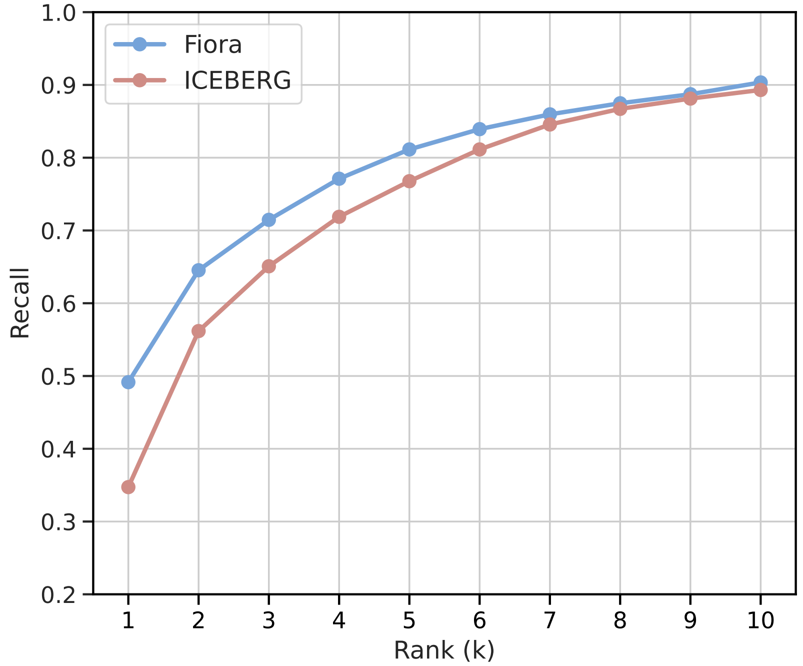

Supplementary Figure 21: Recall at k to retrieve the correct compound by ranking 50 candidates according to spectral similarity, as predicted.

Candidates for positive mode test split compounds were retrieved from PubChem with a mass tolerance of 10 ppm. The MS/MS spectra were predicted by FIORA and ICEBERG and ranked based on cosine similarity to the ground truth spectra. FIORA shows consistently higher recall, in line with the elevated cosine similarity of its spectral predictions.

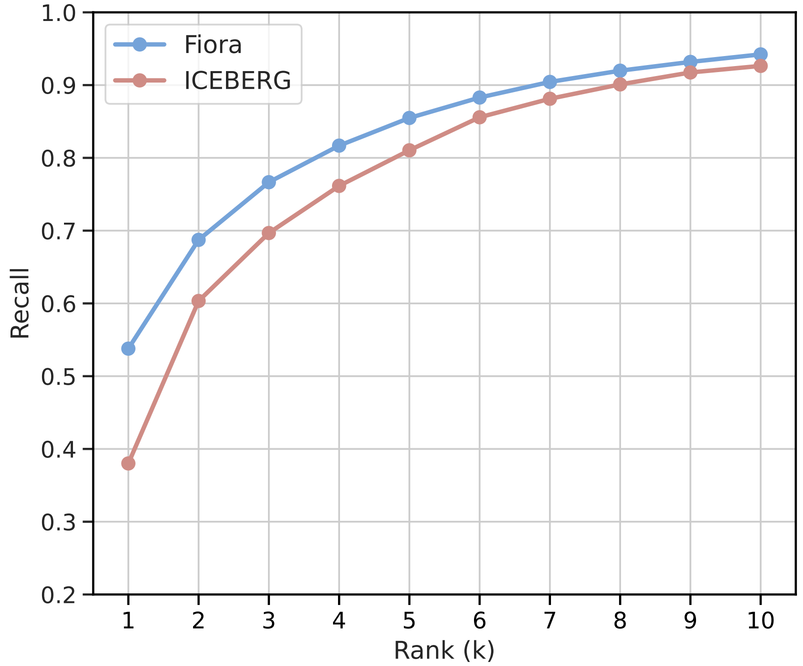

Supplementary Figure 22: Recall at k to retrieve the correct compound by ranking 50 candidates according to spectral similarity without the precursor peak, as predicted.

Candidates for positive mode test split compounds were retrieved from PubChem with a mass tolerance of 10 ppm. The MS/MS spectra were predicted by FIORA and ICEBERG and ranked based on cosine similarity (without the precursor) to the ground truth spectra. FIORA shows consistently higher recall, in line with the elevated cosine similarity of its spectral predictions.

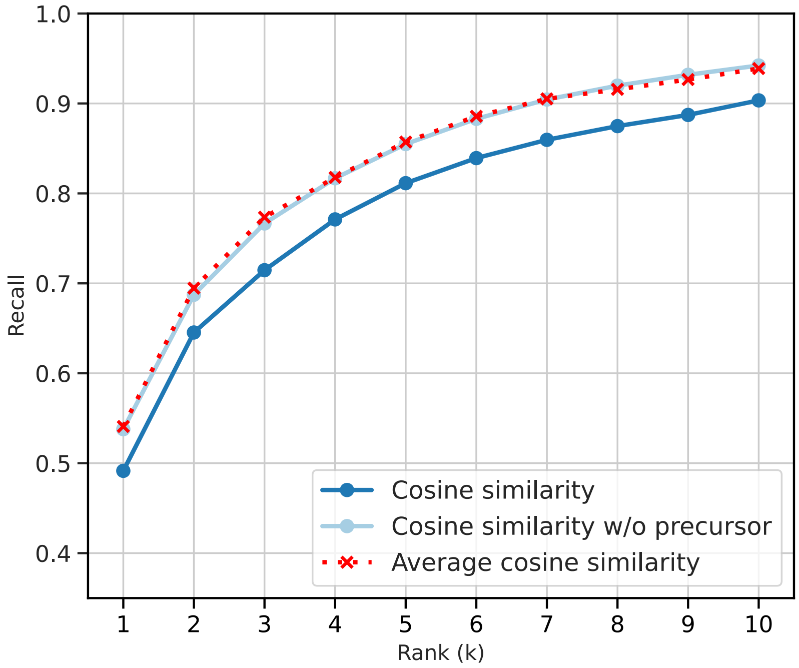

Supplementary Figure 23: Recall at k for different cosine scores, as predicted by FIORA.
The removal of the precursor peaks improves retrieval performance at all ranks. Furthermore, an average cosine score (with and without precursor) seems to perform marginally better, indicating that the inclusion of the precursor peak into a scoring function can improve compound retrieval.

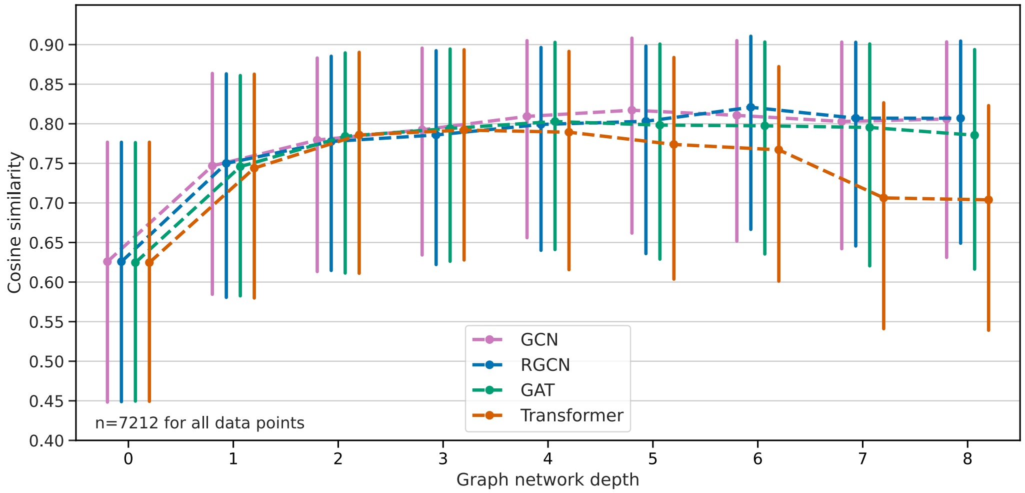

Supplementary Figure 24: Grid search results showing the performance of various graph network architectures with their respective interquartile ranges.
This figure corresponds to Figure 2 in the main manuscript, presenting the median cosine similarity for various GNN architectures, but showing the high variance of the validation scores through the interquartile ranges.

SUPPLEMENTARY TABLES

Supplementary Table 1: Median cosine similarity without precursor peak of spectral predictions to ground truth test spectra. The columns are arranged according to the test sets and precursor ion modes (positive and negative), with the best-performing model highlighted in bold. ICEBERG operates in positive ionization mode only. The † symbol indicates whenever FIORA-OS outperforms all other software.

	Test split +	Test split -	MSnLib +	MSnLib -	CASMI 16 +	CASMI 16 -	CASMI 22 +	CASMI 22 -
Unique compounds	895	437	785	492	381	139	160	98
FIORA	**0.76**	**0.72**	**0.63**	**0.56**	**0.55**	**0.44**	0.25	**0.28**
CFM-ID	0.59	0.43	0.45	0.36	0.48	0.14	0.33	**0.28**
ICEBERG	0.70	-	0.55	-	**0.55**	-	**0.38**	-
FIORA (OS)	0.62	0.63	0.67†	0.65†	0.40	0.48†	0.24	0.30†

Supplementary Table 2: Median cosine similarity without any transformation of spectral predictions to ground truth test spectra. This score does not entail square root transformation of peak intensities. The columns are arranged according to the test sets and precursor ion modes (positive and negative), with the best-performing model highlighted in bold. ICEBERG operates in positive ionization mode only. The † symbol indicates whenever FIORA-OS outperforms all other software.

	Test split +	Test split -	MSnLib +	MSnLib -	CASMI 16 +	CASMI 16 -	CASMI 22 +	CASMI 22 -
Unique compounds	895	437	785	492	381	139	160	98
FIORA	**0.89**	**0.88**	**0.69**	**0.61**	**0.85**	**0.87**	0.21	**0.21**
CFM-ID	0.71	0.56	0.43	0.34	0.82	0.65	**0.28**	0.19
ICEBERG	0.78	-	0.58	-	0.84	-	0.16	-
FIORA (OS)	0.70	0.76	0.78†	0.74†	0.77	0.87†	0.16	0.21†

Supplementary Table 3: Median cosine similarity of [M]+ and [M]- predictions to ground truth spectra from the MSnLib test split.

	[M]+	[M]-
Unique compounds	11	2
Number of spectra	32	3
Cosine similarity with precursor	0.59	0.48
Cosine similarity without precursor	0.48	0.48

Supplementary Table 4: List of molecular and covariate features (on the left), model hyperparameters (center), and training parameters (on the right). Model specifications describe the final set of hyperparameters that were tuned on the validation set.

	Features
Atom	Element
	Num of hydrogens
	Ring type
Bond	Bond type
	Ring type
Covariates	Collision energy
	Molecular weight
	Ionization
	Instrument type

Hyperparameter	Value
Graph network type	RGCN
Graph layers	6
Dense layers	2
Activation function	ELU
Embedding dimension	300
Hidden dimension	300
Input dropout	0.2
Latent dropout	0.1

Training parameter	Value
Num epochs	200
Batch size	256
Learning rate (LR)	0.004
Scheduler	Reduce LR on Plateau (patience = 8; factor = 0.5)

Supplementary Table 5: Overview of the spectral libraries used for training. Sources list only the biggest contributions. Additional information is found on the provider websites `https://www.sisweb.com/software/ms/NIST'17.pdf` `https://systemsomicslab.github.io/compms/msdial/main.html`, and `https://doi.org/10.5281/zenodo.11163381` [5–7].

	Library	**NIST'17**	**MS-Dial**	**MSnLib**
	Publicly available	No	Yes	Yes
	Sources	NIST	Massbank, RIKEN, GNPS, ...	MCEBIO, MCESAF, NIHNP, ...
	RT information	No	Partially	Partially
	CCS values	No	Partially	No
before filtering	Entries	574,826	368,860	177,390
after filtering	MS/MS spectra	54,814	19,587	53,758
	Unique compounds	7,271	4,408	10,341

SUPPLEMENTARY RESULTS

Summary

This Supplementary Information provides additional information on the Fiora model and additional figures and statistics on Fiora's performance. We discuss the impact of collision energies on prediction performance and provide an overview of the dataset with particular focus on the 2022 CASMI challenge. Furthermore, we present example predictions, e.g., Supplementary Figure 2 and Supplementary Figure 3, and statistics providing a broader overview on similarity scores and biases, compound class representations with a particular focus on *lipids and lipid-like molecules*.

Cosine similarity: Distributions and different flavors

As discussed in the main manuscript (Evaluation metrics section), spectral cosine similarity is an effective metric for describing the similarity of two spectra, but places little emphasis on low-intensity fragmentation patterns. To address this shortcoming, cosine similarities are often computed on square root intensities and precursor removal is viable approach to emphasize structure-specific fragment peaks. This section delves more profoundly into the distribution of cosine scores and offers a cross-comparison of the tools based on alternative flavors of cosine similarity.

Supplementary Table 1 shows the median cosine similarity for spectra from which the precursor peak was excluded. This is the equivalent of Table 1 in the main manuscript, but the computation is performed on fragment peaks only. Fiora still outperforms the other tools on all datasets, save for CASMI 22, albeit with a much smaller margin compared to the cosine scores that include the precursor peak. For example, on CASMI 16, the improvement over ICEBERG is less than 0.01. Fiora is more accurate in predicting positive fragment spectra, compared to negative ones. That said, the model continues to demonstrate a notable advantage over CFM-ID in negative ionization mode. ICEBERG is also more accurate than CFM-ID, which exhibits the highest reduction in cosine similarity following precursor removal. The open-source Fiora-OS version performs significantly better in negative mode and on the MSnLib test data, but exhibits decreased performances on the default test split, as well as positive CASMI challenge spectra.

Furthermore, we examine the distributions of cosine similarity with and without the precursor, as illustrated in Supplementary Figure 4. Both Fiora and ICEBERG demonstrate comparable cosine distributions with a pronounced accumulation of high cosine scores (>0.6). However, Fiora exhibits a notable shift towards higher cosine scores compared to ICEBERG, which persists for the non-precursor cosine similarity, albeit to a lesser extent. In contrast, CFM-ID displays a broader distribution, with a greater proportion of average scores. Supplementary Figure 5 depicts the distribution of Fiora's cosine scores, separated according to the test datasets. As with the findings presented in Supplementary Table 1, test split and CASMI 2016 spectra are simulated with predominantly high cosine scores, while MSnLib spectra obtain medium scores and CASMI 2022 spectra obtain low scores.

It is important to note that any alterations to the cosine similarity score will result in a shift in the evaluation process

away from the objective function which prediction models are designed to optimize. This includes the application of a square root transformation to peak intensities. Therefore, we report the unadjusted raw cosine similarity in Supplementary Table 2. Note that this is not the most useful metric for measuring the underlying compound similarity, due to the stark influence exerted by a small number of high-intensity peaks. That said, FIORA continues to demonstrate its superiority across all test sets. The only exception is positive CASMI 22 spectra, which should not be considered canonical, as discussed in detail in the section Data analysis for CASMI 22.

Adducts

The default FIORA model is trained exclusively on [M+H]+ and [M-H]- precursors. However, FIORA's design permits the specification of custom ionization types, as listed under covariates in Supplementary Table 4. To demonstrate this, we added [M]+ and [M]- precursors to the open-source FIORA model (FIORA-OS). Note that the training data was severely limited, comprising 150 [M]+ and 12 [M]- compounds from the MSnLib spectral library. As a result, the cosine similarities, evaluated on the test split compounds (see Supplementary Table 3), are approximately 10 to 20% lower in comparison to the [M+H]+ and [M-H]- predictions. The limited size of the test set, with as few as 2 compounds in the negative mode, renders the result statistically inconclusive and necessitates further investigation. Nevertheless, the model's adaptability to other precursor types is evident. It should be noted that other ionization types, e.g., [M+Na]+, are more challenging to integrate. Given FIORA's focus on local molecular neighborhoods, it is crucial to accurately determine the position of sodium within the molecular structure graph. We propose that this should be addressed algorithmically before passing the molecule to the FIORA model, effectively reducing the prediction task to that of an [M]+ ion, where sodium is included in M.

The impact of collision energy

Collision energy has a profound impact on the probability of bond breaks and, consequently, fragment ion intensities. FIORA explicitly models collision energies as continuous input values, which are integrated into the fragment ion prediction alongside other covariates after the graph convolution layers. In contrast, CFM-ID predicts spectra at three specific energy levels (10, 20, and 40 eV). ICEBERG does not consider collision energies and, as a result, predicts spectra that represent an average for each compound.

This is exemplified by the spectral predictions illustrated in Supplementary Figure 6, Supplementary Figure 7, and Supplementary Figure 8, which depict the fragmentation of the same compound, SNS-032, under varying collision energies. At a collision energy of 20 eV, the precursor ion is clearly present and only three significant peaks are observed. At 30 eV, the precursor is almost entirely degraded and more smaller ions appear in the spectrum. At the high collision setting of 60 eV, the precursor molecule is completely shattered producing an array of many high-intensity low-m/z peaks. Using collision energy as an input feature, FIORA is capable of reproducing peak intensities accurately with a high cosine similarity. However, FIORA struggles to predict the multitude of small fragment ions produced in the high-energy setting. It is probably that this is caused by single-step fragmentation that limits FIORA's output. ICEBERG does not consider collision energies and predicts the same "average" spectrum in all cases. Consequently, the spectral predictions do not specifically match any of the settings and – in this example – exhibit a below average prediction performance. CFM-ID predicts the spectra at 20 and 40 eV and thus is able to somewhat realistically model precursor decay. However, in the case of SNS-032, the spectral predictions of CFM-ID lack accuracy.

Supplementary Figure 9 shows the overall performance of the algorithms at different intervals of collision energies. FIORA's performance declines as the collision energy increases. We suspect that higher collision energies lead to an abundance of higher-order fragments (arising from multiple bond cleavages), which are only partially covered by FIORA's

fragmentation algorithm. This assumption is supported by a similar decline in Fiora's peak intensity coverage, shown in Supplementary Figure 10, at higher collision energy levels. However, ICEBERG exhibits a similar trend to Fiora, with overall lower scores and a smaller decline with increasing collision energy. CFM-ID displays a more rapid decrease in prediction performance, where 10 and 20 eV spectra are predicted with high quality and collision energy levels of 40 eV and above showing significantly worse performance. This indicates that even with multi-step fragmentation, spectra become increasingly harder to predict at higher collision energies. High collision energy spectra are also less prevalent in the training set.

In Supplementary Figure 11 cosine similarity over collision energy is depicted following the exclusion of the precursor peak. Interestingly, the performance remains relatively stable for collision energies between 0 and 60 eV, that is, for Fiora and ICEBERG. It could be concluded that multi-fragmentation takes only a significant effect for very high collision energies, greater than 60 eV. The aforementioned performance loss can therefore be attributed to the diminished role of the precursor peak rather than multi-step fragmentation. Again, ICEBERG shows similar behavior to Fiora , though the performance decline is more subtle.

Supplementary Figure 12 depicts the prediction quality of Fiora for CASMI 16 at different normalized collision energy (NCE) levels. Note that the merged spectrum reflects the experimental setup more closely by modeling the stepped collision energy used in the CASMI 16 challenge, and achieves slightly higher average cosine similarity. Supplementary Figure 13 shows the prediction quality for CASMI 22 split according to NCE. Here, Fiora predicts spectra significantly better for the low collision energy settings. This is discussed in detail in the Data analysis for CASMI 22 section.

Data analysis for CASMI 22

Cosine scores of predicted MS/MS spectra are significantly lower for all algorithms on the CASMI 22 dataset. As noted in the main manuscript, the results must be considered with caution due to inconsistencies between the CASMI 22 data and spectra recorded in the spectral libraries from NIST and MSDial. Specifically, we identified 15 compounds from the CASMI 22 challenge in the initial NIST and MS-Dial spectral libraries, which were subsequently removed for test/training separation. This overlap allows us to examine differences between the spectral measurements recorded in the spectral (training) libraries and the CASMI 22 test set. A considerable number of MS/MS challenge spectra are inconsistent with data from NIST and MS-Dial. For instance, 25% of matching spectra have a cosine similarity of less than 0.1. To clarify, the matching library spectra present a completely valid test set with the same compounds as CASMI 22, but have very little spectral similarity to the actual CASMI 22 spectra, despite similar experimental conditions. With that said, we can specifically utilize the CASMI 22 dataset to investigate differences in the MS/MS data and to examine the limits of the fragmentation algorithms.

Supplementary Figure 15 provides an overview of the MS/MS data in the four test sets. The CASMI 22 spectra were obtained at higher collision energies with a significant portion using 50 eV or higher. Such spectra are barely present in the training data. Note that the 10% test split (first row) gives a good overview of the similar distributions in the training set. As all algorithms exhibit worse performance for high collision energies, this explains the low overall cosine scores for CASMI 22 to some degree. Fiora is also more affected by higher collision energies, which explains why this is indeed its worst performing dataset. This can be explicitly seen in Supplementary Figure 13. At 35 NCE, Fiora predicts spectra at a median cosine similarity of 0.4, which is comparable to that of CFM-ID and ICEBERG. Only at higher NCE values does Fiora's performance decline in comparison. CFM-ID, for example, demonstrates relatively consistent performance across all energy levels.

We also observe a high number of peaks and an abundance of low m/z peaks in the CASMI 22 dataset. In Supplemen-

tary Figure 15, the test split, MSnLib, and CASMI 16 have a maximum peak abundance at around 125 m/z, with a steep drop-off for smaller fragments. In contrast, peak abundance for CASMI 22 reaches its maximum for the smallest recorded peaks at around 50 m/z. Furthermore, peak intensity is split among many peaks for CASMI 22. The right column of Supplementary Figure 15 depicts the number of peaks that account for 80 % of peak intensities. In the case of CASMI 22, intensities are spread among 20 or more peaks for most spectra, indicating a higher number of significant peaks compared to the other datasets. We believe that the high abundance of low m/z peaks, which may cover a substantial amount of the total peak intensity, make CASMI 22 spectra difficult to predict. Interestingly, negative mode spectra have a smaller number of significant peaks, which aligns better with other datasets. This also correlates with an improved performance of FIORA for negative mode CASMI 22 spectra. Overall, the data suggests a considerable structural difference of CASMI 22 spectra compared to the training, test and CASMI 16 spectra. This may also explain the inconsistency between library and CASMI 22 spectra that we reported before.

Most importantly, we observed that algorithms face a severe out-of-distribution problem on CASMI 22. The models learn from the training data, which has a comparable peak m/z distribution to the test split, to distribute the majority of peak intensities among three to five peaks. Consequently, it is unreasonable to expect them to accurately predict spectra where 80% of peak intensities is distributed among 20 or more peaks. It is possible that some form of data pre-processing and noise removal, particularly with regard to low-intensity peaks, could mitigate some of these issues. Importantly though, it is crucial to conduct a thorough data analysis when utilizing any MS/MS prediction software, and it can be concluded that the CASMI 22 dataset is ill-suited for all three algorithms. FIORA is most effective when used in experimental setups that employ low to moderate collision energies, which result in a small number of significant peaks. Stepped collision energies, as seen in CASMI 16, can also be modeled, as FIORA's implementation takes the value of the collision energy as an input.

Mathematical aspects

Let $A = (a_i)$ and $B = (b_i)$ denote two spectra of normalized intensities aligned in the m/z dimension, and suppose in the following that A is the experimental spectrum, whereas B is our prediction. The coverage between experimental and predicted spectrum is defined as

$$\mathrm{cov}(A, B) = \sum_i a_i \mathbb{1}_{b_i > 0} \,.$$

Cosine similarity is typically calculated on the square root of normalized peak intensities. Therefore, we define the cosine similarity as

$$\sqrt{\cos}(A, B) = \frac{\sqrt{A} \cdot \sqrt{B}}{\left\|\sqrt{A}\right\| \left\|\sqrt{B}\right\|}$$

Note that $\left\|\sqrt{A}\right\| = \left\|\sqrt{B}\right\| = 1$ since we have $\sum_i a_i = 1$ and $\sum_i b_i = 1$. It follows that the cosine similarity simplifies to $\sqrt{\cos}(A, B) = \sqrt{A} \cdot \sqrt{B}$.

In general, the cosine similarity reaches it maximum if A and B point into the same direction. Plotting cosine similarity as a function of coverage (Figure 6 in the main manuscript) reveals that it appears to be constrained by a theoretical limit. By fixing a coverage value we restrict B and allow it to only contain non-zero values at certain positions. For instance, if $A = (0.1, 0, 0, 0.2, 0.2, 0, 0.5)$ and we fix a coverage value of 0.4 then by definition B must have non-positive values at the forth and fifth position. Maximizing the coverage therefore results in a constrained optimization problem. More specifically, let I denote the set of indices where B can take non-zero values. We maximize $\sqrt{\cos}(A, B)$ for fixed A and variable B such that $\sum_{i \in I} b_i = 1$ and $b_i = 0$ for $i \notin I$. We easily find that the cosine similarity is maximized for $\hat{B} = (\hat{b}_i)$

where

$$\hat{b}_i = \begin{cases} \frac{a_i}{\sum_{j \in I} a_j} & \text{if } i \in I\,, \\ 0 & \text{if } i \notin I\,. \end{cases}$$

Hence, the maximal cosine value is given by

$$\sqrt{\cos}(A, \hat{B}) = \frac{1}{\sqrt{\sum_{j \in I} a_j}} \sum_{i \in I} a_i\,.$$

Note that if I contains a single element, i.e. $I = \{i\}$, then $\sqrt{\cos}(A, \hat{B}) = \sqrt{a_i}$. Clearly, having the coverage concentrated in a single peak leads to a higher cosine similarity than when the coverage is spread across multiple peaks.

SUPPLEMENTARY REFERENCES

1. Djoumbou Feunang, Y. *et al.* ClassyFire: automated chemical classification with a comprehensive, computable taxonomy. *Journal of cheminformatics* **8,** 1–20 (2016).
2. Lam, H. *et al.* Development and validation of a spectral library searching method for peptide identification from MS/MS. *Proteomics* **7,** 655–667 (2007).
3. Stein, S. E. & Scott, D. R. Optimization and testing of mass spectral library search algorithms for compound identification. *Journal of the American Society for Mass Spectrometry* **5,** 859–866 (1994).
4. Nowatzky, Y., Benner, P., Reinert, K. & Muth, T. Mistle: bringing spectral library predictions to metaproteomics with an efficient search index. *bioRxiv,* 2022–09 (2022).
5. National Institute of Standards and Technology. *NIST17* 2017.
6. Tsugawa, H. *et al.* A lipidome atlas in MS-DIAL 4. *Nature biotechnology* **38,** 1159–1163 (2020).
7. Brungs, C. *et al.* Efficient generation of open multi-stage fragmentation mass spectral libraries (2024).
